# Effectiveness of Physics Forceps in Reducing Dental Pain, Anxiety, and Extraction Efficiency During Mandibular Primary Molar Extraction Among Children Aged 6–9 Years: A Randomized Clinical Trial

**DOI:** 10.1155/ijod/6648179

**Published:** 2026-08-05

**Authors:** Demah Aljarrah, Shadi Azzawi

**Affiliations:** ^1^ Department of Pediatric Dentistry, Faculty of Dentistry, Damascus University, Damascus, Syria, damascusuniversity.edu.sy

**Keywords:** conventional forceps, dental pain and anxiety, physics forceps

## Abstract

**Background:**

Children may experience emotional issues related to tooth extraction because of the forceful intervention, anticipation of pain, sense of loss, and exposure to unpleasant stimuli, which may lead to resistant behaviors in the future. The current study aimed to investigate the effectiveness of physics forceps in alleviating dental pain and anxiety, as well as enhancing procedural efficiency during the extraction of mandibular primary molars in children aged 6–9 years.

**Materials and Methods:**

This randomized, parallel‐group clinical trial enrolled 80 children aged 6–9 years who attended the Department of Pediatric Dentistry, Faculty of Dentistry, Damascus University, and required the extraction of mandibular primary molars. Eligible participants were randomly assigned to two groups: Group I, in which extractions were performed using conventional forceps, and Group II, in which physics forceps were used. Pain was assessed using the face, legs, activity, cry, consolability (FLACC) scale during local anesthesia administration and throughout the extraction procedure. Dental anxiety was evaluated using pulse rate and the facial image scale at baseline, during anesthesia administration, and during extraction. Operative time was recorded in seconds, and extraction‐related complications, including tooth fracture and gingival tearing, were clinically documented.

**Results:**

Intergroup difference was observed in FLACC pain scores during tooth extraction; lower pain scores were recorded in the physics forceps group (*p* = 0.046). Physiological anxiety, assessed by pulse rate, was significantly lower in the physics group during extraction (*p* = 0.002). Similarly, subjective anxiety assessed using the facial image scale was significantly reduced in the physics group during extraction (*p* < 0.05). Extraction time was significantly shorter when physics forceps were used (*p* = 0.001). Although gingival tearing and tooth fracture were less frequent in the physics group, these differences did not reach statistical significance.

**Conclusion:**

The use of physics forceps for mandibular primary molar extraction in children aged 6–9 years significantly reduced dental pain and anxiety and shortened operative time, indicating that it is an effective and child‐friendly alternative to conventional forceps. Although lower rates of gingival tearing and tooth fracture were observed in the physics forceps group, these differences were not statistically significant.

**Trial Registration:**

ClinicalTrials.gov: NCT07366788/2324

## 1. Introduction

Tooth extraction (exodontia) refers to the surgical removal of a tooth from its socket and remains one of the most routinely performed procedures in oral and maxillofacial practice [1]. It is a common yet highly sensitive intervention in the field of pediatric dentistry, as it is frequently accompanied by emotional and behavioral responses that may complicate clinical management [2, 3].

In children, tooth extraction is most commonly indicated in cases of advanced dental caries, irreversibly damaged teeth due to trauma, or as part of orthodontic treatment planning to achieve appropriate space distribution and occlusal balance [2]. Although often technically straightforward, the procedure is frequently perceived by pediatric patients as threatening. Anticipation of pain, perception of applied force, and exposure to an unfamiliar clinical environment can collectively provoke significant fear and anxiety, which may result in uncooperative or avoidant behavior during treatment [4]. Furthermore, the psychological impact of losing a tooth may be perceived by the child as a form of bodily loss or vulnerability [5]. These responses are often intensified by the experience of local anesthesia and the presence of blood, both of which may further reduce cooperation and negatively affect the child’s acceptance of dental care [6, 7].

In response to these challenges, considerable effort has been directed toward improving extraction techniques and minimizing tissue trauma. Among the more recent innovations are physics forceps, introduced by Dr. Richard Golden, which represent a biomechanically driven alternative to conventional forceps. Their mechanism is based on a first‐class lever system that applies a controlled, continuous force to facilitate tooth removal with minimal compressive or torsional stress. Unlike traditional instruments that rely on squeezing and rotational movements, physics forceps are designed to redistribute forces more evenly, thereby reducing localized pressure on the tooth and surrounding alveolar structures and potentially enhancing patient comfort [8, 9].

Evidence from clinical studies supports the use of physics forceps across different extraction scenarios. Hariharan et al. reported reduced intraoperative pain perception in orthodontic premolar extractions using physics forceps, while operative time and postoperative outcomes remained comparable to conventional techniques [10]. Additional studies have suggested that this approach may facilitate simpler extractions with fewer surgical interventions and a lower incidence of complications such as crown fractures, root fractures, and buccal cortical plate damage [11]. Similarly, Patel et al. demonstrated reduced operative time and decreased hard and soft tissue trauma when compared with conventional forceps in premolar extractions [12]. Kapila et al. further confirmed that physics forceps enable atraumatic extractions with improved efficiency and clinical outcomes comparable to traditional methods, alongside a reduction in intraoperative complications [13].

Despite growing evidence in permanent dentition, the application of physics forceps in pediatric dentistry remains relatively limited. Preliminary findings, including those of Elicherla et al., indicate that their use in primary tooth extraction may reduce trauma to supporting tissues and contribute to lower anxiety levels in children during treatment [14].

According to what was mentioned above, this study aimed to evaluate the efficacy of physics forceps in reducing dental pain and anxiety, as well as improving procedural efficiency during the extraction of mandibular primary molars in children aged 6–9 years.

## 2. Materials and Methods

### 2.1. Study Setting and Ethical Considerations

This randomized clinical trial was carried out at the Department of Pediatric Dentistry, Faculty of Dentistry, Damascus University, over 6 months extending from May to December 2025.

Ethical approval for the study was obtained from the Ethics Committee of Damascus University (Approval Number 2324/28042025). All procedures were conducted in accordance with the ethical principles outlined in the Declaration of Helsinki and were reported following the CONSORT recommendations for randomized clinical trials [15, 16].

This retrospective registration resulted from administrative delays encountered during the completion of the registration process. However, the study protocol, including all predefined primary and secondary outcomes, had been finalized and approved before participant recruitment, and no modifications to the primary outcomes were made during the study period.

### 2.2. Participants’ Recruitment and Eligibility

Children attending the Department of Pediatric Dentistry during the study period were screened for eligibility. Of the 85 children initially assessed, 80 fulfilled the inclusion criteria and were subsequently enrolled in the study.

Sample size calculation was performed using 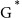
Power software version 3.1.9.4 (Heinrich Heine University, Düsseldorf, Germany). The calculation was based on extraction time as the primary outcome variable, using an estimated effect size (Cohen’s *d*) of 0.609 derived from the study conducted by Elicherla et al. [14]. A statistical power of 80% and a significance level of *α* = 0.05 were adopted. Based on these parameters, the minimum required sample size was calculated to be 34 participants per group. To compensate for potential dropouts, 40 participants were included in each group.

Eligible participants included healthy children aged 6–9 years who required extraction of mandibular primary molars with at least half of the root length remaining. Only cooperative children classified as Frankl behavior rating 3 or 4 were included. Children presenting with systemic diseases, neurological disorders, acute inflammation, active periapical or periodontal infections, or those receiving medications capable of influencing pain perception or bleeding tendency were excluded. Teeth with insufficient remaining root structure that could compromise extraction feasibility were also excluded. Participant selection was performed irrespective of gender, ethnicity, or socioeconomic background.

### 2.3. Blinding and Randomization

Before enrollment, all parents or legal guardians were provided with a detailed explanation of the study procedures, and written informed consent was obtained. Eligible children were then randomly allocated to one of the two study groups using a computer‐generated randomization sequence (www.randomization.com). To ensure balanced group assignment, block randomization with a 1:1 allocation ratio was applied, resulting in two equal groups of 40 participants each.

Allocation concealment was ensured using sequentially numbered, opaque, and sealed envelopes prepared by an independent researcher prior to participant enrollment.

The study did not employ full clinical blinding due to the inherent and clearly visible differences between the conventional forceps and the physics forceps, which made blinding of both the operator and outcome assessors impractical. Participants were also not considered effectively blinded given the nature of the intervention. However, to reduce potential analytical bias, the data analyst was blinded to group allocation throughout the statistical analysis, with all datasets coded before evaluation.

### 2.4. Study Groups

All procedures were explained to the children’s parents or legal guardians, and written informed consent was obtained before recruitment. Subsequently, the participating children were randomly assigned to two groups.

– Group I (control): Extraction of the mandibular primary molars was performed using conventional forceps (inf. 28/6—Bader) (*n* = 40).
– Group II (experimental): Extraction of the mandibular primary molars was performed using physics forceps (DIRECTA Physics) (*n* = 40).

### 2.5. Primary Outcome Measures

• Dental pain:
  1. Face, legs, activity, cry, consolability (FLACC) pain scale: Behavioral observational scale assessing face, legs, activity, cry, and consolability, with total scores ranging from 0 to 10. Measurements were taken at two time points: during local anesthesia administration (*t*_1_) and during tooth extraction (*t*_2_).
• Dental anxiety:
  1. Pulse rate: Objectively measured in beats per minute (bpm) using a pulse oximeter placed on the left index finger at three time points: baseline (*t*_0_), after local anesthesia administration (*t*_1_), and during extraction (*t*_2_).
  2. Facial image scale: Subjectively assessed using a 5‐point pictorial scale ranging from 1 (very happy) to 5 (very unhappy) at three time points: baseline (*t*_0_), after local anesthesia administration (*t*_1_), and after extraction (*t*_2_).

### 2.6. Secondary Outcome Measures

• Extraction duration: Recorded in seconds, measured from the initiation of tooth movement until complete removal.
• Intraoperative complications: Presence or absence of complications, including tooth fracture and gingival tearing, was evaluated clinically.

The FLACC scale was recorded independently by two pediatric dentistry specialists who were trained and calibrated before the commencement of the study to ensure standardized assessment and consistency in scoring. Specifically, the examiners underwent a structured training session before the study, during which they were familiarized with the FLACC scale scoring criteria and standardized assessment procedures. This was followed by a calibration phase in which both examiners independently assessed a set of pilot cases under identical conditions.

Interexaminer reliability was then evaluated using Cohen’s kappa coefficient, which demonstrated good agreement (*κ* > 0.80). The mean of the two examiners’ scores was used for final analysis. The same assessors were also responsible for recording the extraction duration in seconds.

### 2.7. Clinical Procedures

The child’s general medical history was obtained, and comprehensive clinical and radiographic examinations were performed to determine whether the lower primary molars were restorable or required extraction.

Written informed consent was obtained from the parents or legal guardians for both the dental procedure and the child’s participation in the study.

Eligible children were randomly assigned to one of two study groups using a computer‐generated randomization sequence via www.randomization.com.

Dental anxiety was assessed at baseline while the child was seated upright, using both physiological (pulse rate) and subjective (facial image scale) measures. The dental procedure was then explained using the tell–show–do technique.

A topical anesthetic (20% benzocaine: Projel‐20 Topical Anesthetic Gel, Septodont) was applied at the injection site, followed by local infiltration anesthesia using 4% articaine with 1:100,000 epinephrine (Artheek, New Stetic S.A.).

Pain during anesthetic administration was assessed using the FLACC pain scale, and dental anxiety was reassessed immediately after anesthesia onset using the same physiological and subjective measures. The adequacy of anesthesia was confirmed by gentle probing of the targeted tissue.

Tooth extraction was subsequently performed using the assigned extraction instrument:

1. Physics forceps for the experimental group
2. or conventional forceps for the control group.

All extraction procedures were performed by the principal investigator (D.J), an experienced pediatric dentist.

Pain and anxiety levels were recorded during the extraction procedure using the FLACC pain scale and the previously described anxiety assessment tools. The duration of the extraction was measured in seconds from the initiation of tooth removal until complete extraction. Intraoperative complications, including tooth fracture and gingival tearing, were documented. Finally, a sterile gauze pack was placed over the extraction site, and postoperative instructions were provided to the child’s parents or caregivers.

### 2.8. Statistical Analysis

Descriptive statistics, including frequencies, percentages, means, standard deviations, and medians, were calculated.

Normality of distribution was assessed using the Kolmogorov–Smirnov test, which indicated that the data were not normally distributed (*p* < 0.001). Therefore, nonparametric tests were applied for subsequent comparisons.

Differences in gender proportions among the study groups were examined using the Chi‐square test, while age distributions were compared using the Mann–Whitney *U* test.

Intergroup differences in FLACC scores, facial image scale scores, pulse rate, and extraction duration at each procedural time point were analyzed using the Mann–Whitney *U* test.

The Chi‐square test was applied to compare the study groups in terms of the incidence of complications during extraction.

Changes across measurement time points were evaluated using Friedman’s test, with statistically significant results further analyzed by pairwise comparisons using the Wilcoxon signed‐rank test.

All statistical analyses were conducted using SPSS version 25.0 (IBM Corp., Armonk, NY, USA), with statistical significance set at *p*  < 0.05.

## 3. Results

**Figure 1:**
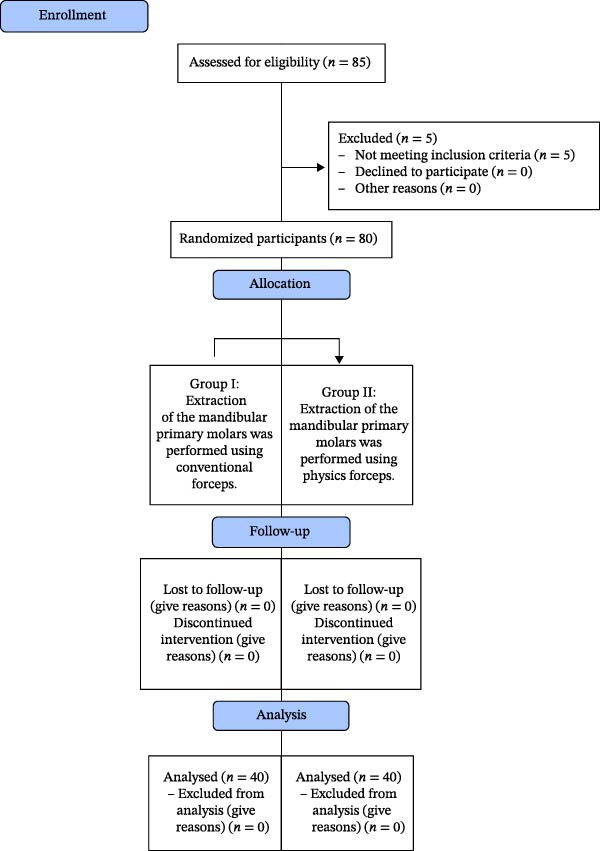
CONSORT diagram.

The CONSORT flow diagram is presented in Figure 1.

A total of 80 children were included, with 40 participants allocated to each group. The conventional group consisted of 16 males (20.0%) and 24 females (30.0%), while the physics group included 22 males (27.5%) and 18 females (22.5%). No statistically significant difference was observed between the two groups regarding gender distribution (*p* = 0.131) (Table 1).

**Table 1:** Gender distribution of the study participants according to the study groups.

Gender	Conventional group		Physics group		Total		*p*‐Value
	*n*	%	*n*	%	*N*	%	
Male	16	20.0	22	27.5	38	47.5	0.131
Female	24	30.0	18	22.5	42	52.5	
Total	40	50.0	40	50.0	80	100.0	

Moreover, each study group comprised an equal distribution of tooth type, including 20 first primary mandibular molars and 20 s primary mandibular molars.

Table 2 presents that the age of children in the conventional group ranged from 6 to 9 years, with a mean age of 8.05 ± 0.93 years and a median of 8.00 years. No statistically significant difference was observed between the two groups with respect to age distribution (*p* = 0.807).

**Table 2:** Age distribution of participants in the conventional and physics forceps groups.

Study groups	Minimum	Maximum	Median	Mean	Std. deviation	*p*‐Value
Conventional	6.00	9.00	8.00	8.05	0.932	0.807
Physics	7.00	9.00	8.00	7.98	1.000	
Total	6.00	9.00	8.00	8.01	0.961	—

Intergroup comparisons (Table 3) revealed no significant difference in FLACC scores between the conventional and physics forceps groups during local anesthesia administration (*t*_1_) (*p* = 0.574). However, during tooth extraction (*t*_2_), the physics forceps group exhibited significantly lower FLACC scores compared to the conventional group (*p* = 0.046), indicating reduced procedural pain.

**Table 3:** Intergroup comparison of FLACC, facial image scale, pulse rate, and extraction duration at different time points using the Mann–Whitney *U* test.

FLACC							
	Conventional group			Physics group			*p*‐Value
	Mean	Std. deviation	Median	Mean	Std. deviation	Median	
*t*_1_	3.05	1.037	3.00	3.18	1.483	3.00	0.574
*t*_2_	2.70	0.758	3.00	2.25	1.645	2.00	0.046 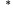
Pulse rate							
*t*_0_	96.50	19.965	97.50	94.15	17.565	95.00	0.544
*t*_1_	105.73	16.204	105.00	104.70	17.539	105.00	0.806
*t*_2_	110.70	14.708	108.00	99.43	15.257	100.00	0.002 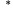
Facial image scale							
*t*_0_	1.90	0.709	2.00	1.58	0.712	1.00	0.060
*t*_1_	2.65	0.770	3.00	2.80	0.758	3.00	0.450
*t*_2_	3.38	1.030	3.00	1.85	1.001	2.00	0.001 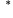
Extraction duration							
	25.98	10.408	24.00	12.43	3.249	11.50	0.001 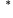

*Note:* (*t*_0_): baseline, (*t*_1_): during/after local anesthesia administration, and (*t*_2_): during/after extraction.

^∗^Statistically significant difference (*p*‐Value < 0.05).

Pulse rate was similar between groups at baseline (*t*_0_) and after local anesthesia administration (*t*_1_) (*p* > 0.05), but during extraction (*t*_2_), the physics group showed a significantly lower mean pulse rate than the conventional group (*p* = 0.002), reflecting a lower physiological stress response (Table 3).

Dental anxiety, assessed using the facial image scale, demonstrated intergroup differences. The physics forceps group showed a nonsignificant trend toward lower anxiety at baseline (*t*_0_; *p* = 0.060) and a significant reduction during extraction (*t*_2_; *p* = 0.001), whereas no significant difference was observed after anesthesia administration (*t*_1_; *p* = 0.450). Moreover, extraction duration was significantly shorter in the physics forceps group (12.43 ± 3.25 s) compared to the conventional group (25.98 ± 10.41 s, *p* = 0.001) (Table 3).

Table 4 shows that gingival tearing was observed in 10 cases (12.5%) overall, including seven cases (8.8%) in the conventional group and three cases (3.8%) in the physics group. However, this difference was not statistically significant (*p* = 0.155). Also, a tooth fracture occurred in three cases (3.8%), all of which were recorded in the conventional group, while no cases were observed in the physics group. Nevertheless, the difference between the two groups did not reach statistical significance (*p* = 0.120).

**Table 4:** Comparison of intraoperative complications between the conventional and physics forceps groups.

Incidence of gingival tearing							
Incidence	Total		Conventional group		Physics group		*p*‐Value
	*N*	%	*N*	%	*N*	%	
Yes	10	12.5	7	8.8	3	3.8	0.155
No	70	87.5	33	41.2	37	46.2	
Incidence of tooth fracture							
Yes	3	3.8	3	3.8	0	0.0	0.120
No	77	96.2	37	46.2	40	50.0	

Intragroup analysis using Friedman’s test (Table 5) indicated that pulse rate and facial image scale scores varied significantly across the procedural stages within each group (*p* = 0.001). Pairwise comparisons with the Wilcoxon signed‐rank test confirmed that all time‐point differences (*t*_0_ vs. *t*_1_, *t*_0_ vs. *t*_2_, and *t*_1_ vs. *t*_2_) were statistically significant, demonstrating dynamic changes in anxiety and physiological response during the procedure. Similarly, intragroup comparison of FLACC scores (Table 6) showed a significant decrease in pain from *t*_1_ to *t*_2_ within both groups (conventional: *p* = 0.040; physics: *p* = 0.002), indicating that pain perception decreased from the anesthetic administration phase to the extraction phase.

**Table 5:** Friedman test for comparing the facial image scale and pulse rate scores across time points within each study group.

Study groups	*T*_0_		*T*_1_		*T*_2_		*p*‐Value
	Mean ± SD	Median	Mean ± SD	Median	Mean ± SD	Median	
Pulse rate							
Conventional	96.50 ± 19.965	97.50	105.73 ± 16.204	105.00	110.70 ± 14.708	108.00	0.001 ^∗^
Physics	94.15 ± 17.565	95.00	104.70 ± 17.539	105.00	99.43 ± 15.257	100.00	0.001 ^∗^
Facial image scale							
Conventional	1.90 ± 0.709	2.00	2.65 ± 0.770	3.00	3.38 ± 1.030	3.00	0.001 ^∗^
Physics	1.58 ± 0.712	1.00	2.80 ± 0.758	3.00	1.85 ± 1.001	2.00	0.001 ^∗^

*Note:* (*t*_0_): baseline, (*t*_1_): during/after local anesthesia administration, and (*t*_2_): during/after extraction.

^∗^statistically significant difference (*p*‐Value < 0.05).

**Table 6:** Intragroup comparison of FLACC pain scores between procedural time points using the Wilcoxon signed‐rank test.

FLACC					
	*T*_1_		*T*_2_		
	Mean ± SD	Median	Mean ± SD	Median	
Conventional	3.05 ± 1.037	3.00	2.70 ± 0.758	3.00	0.040 ^∗^
Physics	3.18 ± 1.483	3.00	2.25 ± 1.645	2.00	0.002 ^∗^

*Note:* (*t*_1_): during/after local anesthesia administration and (*t*_2_): during/after extraction.

^∗^Statistically significant difference (*p*‐Value < 0.05).

## 4. Discussion

The objective of the present study was to assess the clinical performance of physics forceps in alleviating pain and dental anxiety while enhancing procedural efficiency during mandibular primary molar extractions among children aged 6–9 years.

Although pediatric dental care is typically provided in a safe and nonthreatening clinical setting, many children continue to perceive dental treatment as painful and distressing. Such perceptions vary widely, ranging from complete comfort to pronounced discomfort, and are influenced by both the administration of local anesthesia and the complexity of the dental procedure [17].

Ghanei et al. reported that nearly one‐third of dental visits were perceived as painful. Furthermore, tooth extraction was the most painful procedure in 62.4% of cases, whereas restorative procedures were less frequently associated with pain (38.8%). The study concluded that injections during local anesthesia and tooth extraction are the main contributors to pain during dental treatment [3].

Impaction, extensive caries, root or crown fractures, and advanced periodontal disease are common indications for tooth extraction, which is considered a routine dental procedure [18].

Safe, efficient, and predictable removal can be achieved by adhering to proper extraction techniques and biomechanical principles, thereby minimizing complications. Numerous studies have advocated the application of physics forceps during tooth extraction as a means to enhance the clinical efficacy of the procedure, mitigate potential complications, and alleviate both pain and anxiety [19].

In pediatric patients, the use of physics forceps has been proposed as a technique that can reduce procedural time, decrease pain and anxiety, and limit iatrogenic trauma to both the child and the clinician, offering a more controlled and atraumatic approach to mandibular primary molar extractions [14]. However, there is a massive need for more studies to evaluate the efficacy of the physics forceps among children, especially in terms of pain and anxiety.

The participants in this study were children between 6 and 9 years of age. This age range was selected because children at this stage generally exhibit better cooperation during dental treatments, can provide dependable self‐reports of pain and anxiety using standardized assessment tools, and can follow structured instructions in clinical procedures [20].

Dental anxiety was evaluated using both physiological and subjective measures, namely, pulse rate and the facial image scale. The pulse rate, a well‐established indicator of autonomic arousal, is regulated by the sympathetic nervous system and reflects the individual’s level of stress and anxiety [21, 22].

Subjectively, the facial image scale is a recognized self‐report tool used to assess fear and anxiety in children. It comprises five pictorial representations of facial expressions, spanning from a highly positive (score 1) to a highly negative expression (score 5). Lower scores indicate minimal or no anxiety, whereas higher scores correspond to pronounced anxiety [23, 24].

Pain was measured using the FLACC scale, which is a behavioral tool designed to evaluate pain in children by observing five specific domains: facial expression, leg movement, activity level, crying, and consolability. Each domain is assigned a score from 0 to 2, producing a cumulative score between 0 and 10, where 0 reflects no observable pain and 10 denotes the highest level of pain [25, 26].

While self‐report methods are generally regarded as the primary approach for pain assessment in older children due to their ability to directly communicate subjective experiences, observational tools such as the FLACC scale remain valuable as complementary or backup measures [27, 28].

In the present study, 4% articaine was administered using the buccal infiltration technique rather than an inferior alveolar nerve block with 2% lidocaine.

This approach was justified by the distinctive physicochemical characteristics of articaine, which, although comparable to other local anesthetics, contains a thiophene ring instead of the conventional benzene ring. This structural modification enhances its liposolubility and significantly improves tissue diffusion, allowing for more effective penetration into mandibular bone and surrounding tissues [29]. Previous studies have consistently demonstrated the efficacy of articaine infiltration for pulp therapy and tooth extraction in pediatric patients [30, 31].

In contrast, the inferior alveolar nerve block is associated with several limitations in children, including anesthesia of an extensive region, prolonged soft tissue numbness, and an increased risk of postoperative soft tissue injury. Consequently, there has been growing clinical interest in utilizing articaine infiltration as a safer and more targeted alternative for achieving adequate anesthesia of mandibular molars in pediatric dentistry [32].

In the present study, the FLACC scale revealed a significant difference in pain perception between conventional and physics forceps, with the latter group demonstrating lower mean pain scores.

This finding can be attributed to the biomechanical design of physics forceps, which function as specialized dental extractors rather than traditional forceps. By employing first‐class lever mechanics and exploiting the phenomenon of creep, where the bone and the periodontal ligament gradually deform under sustained load, these instruments enable controlled, minimally traumatic extractions [33]. Such an approach not only reduces procedural pain in pediatric patients but may also limit the development of negative dental experiences, thereby potentially decreasing dental fear and anxiety among peers [14].

These observations are in line with previous reports [10, 14, 34, 35]. Supporting evidence from the literature further underscores these findings: Shetty and Sharma reported that while most children in the conventional group experienced mild to severe pain, participants in the physics forceps group largely reported no pain [36]. Similarly, Hariharan et al. documented lower mean pain scores in the physics group (0.6 ± 0.6) compared to the conventional group (1.04 ± 0.9) [10], a trend also confirmed by Patel et al. [12]. In pediatric patients, Elicherla et al. observed a comparable pattern, with the physics forceps group recording a mean pain score of 0.60 ± 0.90, markedly lower than the 4.82 ± 1.50 observed in the conventional group [14].

Moreover, this study demonstrated a significant difference in dental anxiety between the experimental and control groups, as measured both physiologically (pulse rate) and subjectively (facial image scale). During extraction, the control group exhibited higher anxiety levels (pulse rate = 110.70 ± 14.71; FIS = 3.38 ± 1.03) compared to the experimental group (pulse rate = 99.43 ± 15.26; FIS = 1.85 ± 1.00).

Several factors may account for this disparity. First, the biomechanical design of physics forceps, previously described, facilitates controlled, minimally traumatic extractions, thereby reducing pain and procedural stress [33].

Second, the close interplay between pain and anxiety provides a plausible explanation. Painful or prolonged nociceptive stimuli can activate stress‐related neurophysiological pathways, including the release of cortisol and other stress hormones, which in turn amplify emotional distress and anticipatory anxiety [22, 37, 38]. This bidirectional relationship, as highlighted by the American Psychological Association [22] and supported by Bchara et al. [39], illustrates how heightened pain can reinforce anxiety, creating a cycle of increased emotional and sensory sensitivity.

Third, the reduction in intraoperative duration afforded by the physics forceps contributes to improved behavioral management. Shorter appointments are known to enhance cooperation and compliance among pediatric patients [40, 41].

This observation aligns with the findings of Elicherla et al. [14], who reported a lower mean rating mood score in the physics group (2.86 ± 1.08) compared to the conventional group (3.12 ± 1.01), reflecting reduced procedural anxiety and improved patient experience.

In the present study, the use of physics forceps was associated with a marked reduction in extraction time (25.98 ± 10.408 vs. 12.43 ± 3.249: *p* = 0.001). This efficiency can be attributed to their biomechanical design, which employs a first‐class lever to apply controlled and gradual force, minimizing dependence on the clinician’s manual strength and facilitating rapid, atraumatic tooth removal [36].

Comparable findings have been reported in the literature. Kapila et al. [13] observed that extractions with conventional forceps required an average of 29.30 s, whereas physics forceps reduced the mean time to 24.34 s. Similarly, Patel et al. [12] reported shorter operating times with physics forceps (58.8 ± 48.13) compared to conventional forceps (88.33 ± 37.59). Shetty and Sharma [36] documented an even more pronounced difference, with a mean extraction time of 10.7 ± 13.6 s in the physics group versus 60.3 ± 68.0 s in the conventional group. Likewise, Elicherla et al. [14] reported mean extraction times of 11.85 ± 3.77 s for physics forceps and 33.98 ± 12.29 s for conventional forceps.

A small number of cases of gingival tearing (3/40) were observed in the experimental group, while no instances of tooth fracture were reported. This finding contrasts with the results of Elicherla et al. [14], who documented a significant difference between groups with respect to tooth fracture. Such discrepancies may be attributed to methodological differences between the studies, particularly variations in the age range of the participants, which can influence tissue resilience and the risk of iatrogenic injury during extraction.

Clinically, the findings of the present study suggest that the use of physics forceps for mandibular primary molar extractions in children aged 6–9 years may offer several advantages over conventional forceps, including significant reductions in procedural pain and dental anxiety, as well as shorter operative times. Finally, although lower frequencies of gingival tearing and tooth fracture were observed in the physics forceps group; however, these differences did not reach statistical significance.

The present study has several limitations. First, the participant age range was limited to 6–9 years, which restricts the generalizability of the findings to younger or older pediatric populations. Second, the assessment of dental pain relied partly on objective measures, which may not fully capture individual perception or emotional responses. Third, postoperative follow‐up was confined to the immediate period surrounding the extraction, preventing evaluation of longer‐term effects on children’s dental anxiety. Finally, the study focused exclusively on mandibular primary molars, and the results may not be directly applicable to maxillary teeth.

## 5. Conclusion

The findings of the current study revealed that the use of physics forceps for the extraction of mandibular primary molars in children aged 6–9 years was associated with a significant reduction in dental pain and anxiety, as well as a shorter operative time. Although lower rates of gingival tearing and tooth fracture were observed in the physics forceps group, these differences were not statistically significant.

Collectively, these outcomes suggest that physics forceps represent an effective and child‐friendly alternative to conventional extraction methods, enhancing procedural efficiency while improving patient comfort and fostering a more positive dental experience in pediatric patients.

**Nomenclature**
FIS: Faces image scale.

## Author Contributions

**Demah Aljarrah:** conceptualization, methodology, investigation, data curation, formal analysis, writing – original draft. **Shadi Azzawi:** supervision, validation, methodology, writing – review and editing.

## Funding

This study did not receive any specific funding.

## Disclosure

All authors have read and approved the final version of the manuscript. The corresponding author (Demah Aljarrah) had full access to all the data in this study and takes complete responsibility for the integrity of the data and the accuracy of the data analysis.

## Ethics Statement

Ethical approval was obtained from the Ethics Committee of Damascus University (Approval Number 2324/28042025). Written informed consent was obtained from parents or legal guardians before participation.

## Conflicts of Interest

The authors declare no conflicts of interest.

## Data Availability

The datasets generated during and/or analyzed during the current study are available from the corresponding author upon reasonable request.
